# The Spike Protein of SARS-CoV-2 Impairs Lipid Metabolism and Increases Susceptibility to Lipotoxicity: Implication for a Role of Nrf2

**DOI:** 10.3390/cells11121916

**Published:** 2022-06-14

**Authors:** Vi Nguyen, Yuping Zhang, Chao Gao, Xiaoling Cao, Yan Tian, Wayne Carver, Hippokratis Kiaris, Taixing Cui, Wenbin Tan

**Affiliations:** 1 Department of Cell Biology and Anatomy, School of Medicine, University of South Carolina, Columbia, SC 29209, USA; vi.nguyen@uscmed.sc.edu (V.N.); zhangyuping@csu.edu.cn (Y.Z.); chao.gao@uscmed.sc.edu (C.G.); caoxiao410@gmail.com (X.C.); tianyancs@csu.edu.cn (Y.T.); wayne.carver@uscmed.sc.edu (W.C.); taixing.cui@uscmed.sc.edu (T.C.); 2 Department of General Surgery, The 3rd Xiangya Hospital of Central South University, Changsha 410013, China; 3 Department of Obstetrics and Gynecology, Xiangya Hospital of Central South University, Changsha 410008, China; 4 Department of Biomedical Engineering, College of Engineering and Computing, University of South Carolina, Columbia, SC 29208, USA; 5 Drug Discovery & Biomedical Sciences, College of Pharmacy, University of South Carolina, Columbia, SC 29208, USA; kiarish@cop.sc.edu

**Keywords:** spike protein, SARS-CoV-2, lipid metabolism, lipotoxicity, autophagy, ferroptosis

## Abstract

Coronavirus disease 2019 (COVID-19) patients show lipid metabolic alterations, but the mechanism remains unknown. In this study, we aimed to investigate whether the Spike protein of severe acute respiratory syndrome coronavirus 2 (SARS-CoV-2) impairs lipid metabolism in host cells. We generated a Spike cell line in HEK293 using the pcDNA vector carrying the Spike gene expression cassette. A control cell line was generated using the empty pcDNA vector. Gene expression profiles related to lipid metabolic, autophagic, and ferroptotic pathways were investigated. Palmitic acid (PA)-overload was used to assess lipotoxicity-induced necrosis. As compared with controls, the Spike cells showed a significant increase in lipid depositions in cell membranes as well as dysregulation of expression of a panel of molecules involving lipid metabolism, autophagy, and ferroptosis. The Spike cells showed an upregulation of nuclear factor erythroid 2-related factor 2 (Nrf2), a multifunctional transcriptional factor, in response to PA. Furthermore, the Spike cells exhibited increased necrosis in response to PA-induced lipotoxicity compared to control cells in a time- and dose-dependent manner via ferroptosis, which could be attenuated by the Nrf2 inhibitor trigonelline. We conclude that the Spike protein impairs lipid metabolic and autophagic pathways in host cells, leading to increased susceptibility to lipotoxicity via ferroptosis which can be suppressed by a Nrf2 inhibitor. This data also suggests a central role of Nrf2 in Spike-induced lipid metabolic impairments.

## 1. Introduction

Coronavirus disease 19 (COVID-19) is a pandemic viral infection that threatens global public health since the initial outbreak in December 2019 at the epicenter of Wuhan City, Hubei Province, China [1]. COVID-19 is caused by the severe acute respiratory syndrome coronavirus 2 (SARS-CoV-2) with high pathogenicity and contagiousness [2]. SARS-CoV-2 is a positive-sense single-stranded RNA virus that is capable of infecting human beings, together with six other coronaviruses [2]. SARS-CoV-2 is assumed to be zoonotic and shares 96.3% sequence identity with the bat coronavirus RaTG13 [3]. The angiotensin converting enzyme 2 (ACE2) is the receptor mediated virus entry into host cells via the SARS-CoV-2 Spike protein. Cleavage of Spike protein by Furin and Transmembrane Serine Protease 2 (TMPRSS2) facilitates SARS-CoV-2 entry into host cells [4]. ACE2 and TMPRSS2 are the host determinants during initial infection [5]. Neuropillin (NRP) 1 has been identified as an additional host factor to facilitate SARS-CoV-2 entry upon cleavage by Furin [6,7,8]. In addition, several intracellular pathological effects of the Spike have been reported. For example, The Spike upregulates expression of the hemeoxygenase-1 (HO-1) in kidney cell lines [9]. The Spike protein can cause downregulation of ACE2 and impair endothelial mitochondria functions [10]. Expression of the Spike protein subunit 1 in lung epithelial cells can result in natural killer cell-reduced degranulation [11].

COVID-19 patients can be asymptomatic or symptomatic. The mortality rate of COVID-19 varies in different geographic locations and patient populations [1]. More severe symptoms have been experienced by the patients with metabolic-associated preconditions such as hypertension, cardiovascular disorders (CVD), obesity, and diabetes mellitus (DM) [12]. One common pathogenic co-factor related to hypertension, obesity, DM, and CVD is hypercholesterolemia. Accumulated evidence shows that COVID-19 directly interplays with dire cardiovascular complications including myocardial injury and heart failure, resulting in elevated risk and adverse outcomes among infected patients [13]. COVID-19-associated cardiac complications may become even worse in the setting of cardiometabolic pathologies associated with obesity, although obesity per se is a strong risk factor for severe COVID-19 [14]. Our recent studies have shown decreased levels of total cholesterol (TC), low density lipoprotein cholesterol (LDL-c) and high-density lipoprotein cholesterol (HDL-c) in COVID-19 patients, which are associated with disease severity and mortality [15,16,17]. Mechanistically, lipids have been shown to be a critical contributor to transmission, replication, and transportation for some types of viruses. For example, lipid rafts have been reported to be necessary for SARS virus replication [18]. Although it has been firmly established that obesity and obesity-related complications are major risk factors for COVID-19 severity, the underlying mechanisms have yet to be determined.

Ferroptosis is an iron-dependent lipid peroxidation-driven cell death. During ferroptosis, acyl-CoA synthetase long-chain family 4 (Ascl4)-dependent lipid biosynthesis regulates the function of the lipoxygenase for lipid peroxidation [19]. Ferritin heavy chain 1 (Fth1) functions as iron storage and has ferroxidase activity [20,21]. Ferroptosis can directly promote cellular inflammation via upregulation of prostaglandin E synthase 2 (PTGS2) [22]. Emerging evidence has shown that multi-organelles involve in ferroptosis including lysosome, mitochondria, endoplasmic reticulum, peroxisomes, and Golgi apparatus where the oxidative stress, lipid synthesis and peroxidation, and oxidated cargo sorting and processing occur [19]. In particular, autophagy, which is regulated by lipid metabolism, interplays with and promotes ferroptosis [20]. Formation of autophagosomes with engulfed cargo for degradation requires autophagy-related genes (ATGs)-related ubiquitin-like reaction and subsequent microtubule-associated protein 1 light chain 3 beta (LC3)-involved ubiquitin-like reaction [23]. A lipidated LC3, e.g., LC3 II, resulting from the proteolytic cleavage of LC3, is associated with autophagosomes, which have been widely used for monitoring autophagic flux process [20,21].

In this study, we attempted to explore the direct interplays among the Spike protein, lipid metabolism, autophagy, and ferroptosis in host cells. We found that the Spike protein impairs lipid metabolic and autophagic pathways in host cells, leading to increased susceptibility to lipotoxicity most likely via switching on nuclear factor erythroid 2-related factor 2 (Nrf2)-mediated ferroptosis.

## 2. Methods

### 2.1. Materials

Human embryonic kidney 293 cells (HEK293) and DMEM medium were purchased from ATCC (ATCC, Manassas, VA, USA). The SARS-CoV-2 S gene (GenBank: QHU36824.1) in fusion with a His tag at c-terminal was synthesized and cloned into a pcDNA3.1 vector (Genscript, Piscataway, NJ, USA). The coding sequence was optimized for expression in human cells [24]. The vector was referred as pcDNA-Spike with a detailed description in our previous report [24].

Anti-His tag and LC3 antibodies were purchased from Millipore Sigma (Millipore Sigma, Burlington, MA, USA). Anti-Adipose Differentiation-Related Protein (ADRP, or Perilipin-2, PLIN2), Nrf2, PTGS2, and phosphoinositide-3-Kinase (PI3K)-beta antibodies were obtained from ProteinTech (ProteinTech, Rosemont, IL, USA). Anti-ATG7 antibody was purchased from Abcam (Abcam, Waltham, MA, USA). Anti-scavenger receptor class B type 1 (SRB1) antibody was obtained from Novus (Novus, Centennial, CO, USA). Anti-Fth1, HRP-anti-rabbit or mouse secondary antibodies, and radioimmunoprecipitation assay (RIPA) lysis buffer were obtained from Santa Cruz Biotech (Santa Cruz Biotech., Inc., Dallas, TX, USA). Primers for real time RT-PCR were synthesized by IDT (IDT, Coralville, IA, USA) (Supplementary Table S1). RNA extraction kit, RT kit, and SYBR green master mix was obtained from Zymo Research (Zymo, Irvine, CA, USA), Takara Bio USA (Takara Bio, Mountain view, CA, USA), and Bio-Rad (Bio-Rad, Hercules, CA, USA), respectively.

### 2.2. Generation of the Spike-Protein Stable Expression Cell Line

The HEK293 cells were grown in Dulbecco’s Modified Eagle’s medium (DMEM) containing 10% fetal bovine serum (FBS). The pcDNA-Spike or pcDNA vector was transfected into the cells using lipofectamine 300 reagent (ThermoFisher, Waltham, MA, USA). Two days after transfection, the cells were treated by G418 starting from the concentration of 100 µg/mL with a gradual increase to 800 µg/mL during the following 2 weeks. The individual colonies with stable integration of the pcDNA-Spike (HEK_Spike) or pcDNA vector (HEK_pcDNA) were selected and expanded. The HEK_Spike stable colonies were confirmed to express the Spike protein using immunoblot. The cells were maintained in DMEM with 10% FBS regularly for further experiments.

### 2.3. Lipid (Oil Red O) Staining

The Lipid (Oil Red O) kit was obtained from Millipore Sigma. The HEK293, HEK_pcDNA, and HEK_Spike cells were fixed by 10% formalin and followed by 60% isopropanol treatment, followed by an incubation of the Oil Red O working solution for 15 min. The cell nucleus was counterstained using hematoxylin. The images were acquired using an ImagXpress Pico Automated system (Molecular Device, San Jose, CA, USA). Oil Red O stain was extracted in isopropanol followed by a measurement of absorbance at 492 nm using a Thermo Scientific Multiskan Spectrophotometer system.

### 2.4. Real Time-RT PCR and Western Blot

RT was performed in a 20-µL reaction containing 1.0~5 µg of total RNA, 0.5 mM dNTPs, 0.5 µg of oligo (dT) 15-mer primer, 20 units of RNasin, and 5 units of SMART Moloney murine leukemia virus reverse transcriptase) in 1x RT buffer (Clontech, Mountain View, CA, USA) at 42 °C for 2 h. A converted index for three reference genes were used to normalize the amplification data: GAPDH, Nono, and β-actin. Expression levels of a panel of 83 genes related to lipid metabolic, autophagic, and ferroptotic pathways (Supplementary Table S1) were determined using real time RT-PCR. Briefly, the real time PCRs reactions were carried out in a 25-µL total volume containing 10 ng of each cDNA template and 10 pmol of each specific primer in 1 × SYBR Green qPCR Master Mix (Bio-Rad) with a duplication of each reaction. The PCR parameters include one cycle of 95 °C for 2 min and 45 cycles of 95 °C for 15 s at and 60 °C for 60 s. The cycle number at which fluorescence crossed the cycle threshold (*Ct*) for the target and reference genes was used to evaluate the amplification efficiency for the relative quantification of the real time PCR.

For Western Blot assay, cellular proteins were extracted from HEK, HEK_pcDNA, or HEK_Spike cells using RIPA lysis buffer (Santa Cruz Biotech., Inc.), separated by SDS-PAGE, followed by a transfer onto PVDF membranes. Primary antibodies were added and followed by HRP-labeled secondary antibodies. Images were acquired using a Bio-Rad Gel Imaging System.

### 2.5. Palmitic Acid (PA)-Induced Lipotoxicity Assay

The HEK293, HEK_pcDNA, and HEK_Spike cells were cultured in a 96-well plate and reached to 80% confluence on the next day before treatment. The cells were kept in DMEM medium with 5% FBS during the entire treatment procedure. The cells were treated with PA (free BSA) in various concentrations from 250 to 1000 µM for 24, 48, or 72 h. For treatment control groups, the cells were treated by the same concentrations of BSA in parallel. For the inhibitory assay, the cells were pre-incubated by autophagic inhibitors (125 µM trigonelline (TRG), or 10 µM Wortmannin), or a ferropotosis inhibitor (ferrostatin, 2 µM), or an apoptosis inhibitor (necrostatin-1, 10 µM) in DMEM medium with 5% FBS for 2 h, followed by 750 µM PA treatment for 24 h. The cells were then co-stained by Propidium Iodide (PI) and Hoechst 33342 NucBlue Live Cell Stain dye (ThermoFisher) to show the dead and live cells. The dyes were excited at 535 nm and 405 nm, respectively. Images were acquired and cell viability analyses were performed using an ImagXpress Pico Automated system.

### 2.6. Viral Production and H9C2 Cell Culture

The Spike gene was cleaved from pcDNA-Spike plasmid and cloned into lentiviral vector pLV-mCherry (Addgene, Watertown, MA, USA) with removal of mCherry gene to generate pLV-Spike plasmid. The lentiviral production followed our previous report with slight modifications [24]. We generated a Spike-pseudotyped (Spp) lentivirus with the Spike protein as the viral surface tropism as well as expression of the Spike protein, which was referred to as Cov-Spp-S virus. Briefly, Phoenix cells (ATCC) were cultured in DMEM containing 10% FBS and transfected by pLV-Spike using a calcium phosphate kit (ThermoFisher). The control virus with VSV-G as the tropism and expression of mCherry was generated by co-transfection of pLV-mCherry and pMD2.G vector (Addgene) into the Phoenix cells, which was referred to as VSV-G virus. Seventy-two hours post transfection, the supernatant containing released virus was collected, clarified by centrifuging at 5000× *g* for 15 min, passed through a 0.45 µm filter disk, followed by ultracentrifugation at 24,000 rpm for 2 h using Beckman SW41 rotor. The precipitated virus was resuspended in cold PBS buffer, aliquoted, and stored at −80 °C before use. The viral titer was to quantify the RNA copies of the Spike or mCherry using a real time RT-PCR assay.

H9C2 cells (ATCC) were cultured in DMEM (10% FBS) medium in a 96 well-plate with 80% confluence. The cells were infected twice at the first and second days using Cov-Spp-S or VSV-G lentivirus (4.8 × 10^7^ particles per well) with addition of polybrene (1 µg/mL); the cells were then incubated for an additional 5 days, followed by treatment with PA (675 µM) for 24 h in DMEM medium containing 5% FBS. The same concentration of BSA was used for treatment control groups in parallel. For the inhibitory assay, the cells were pre-incubated with 125 µM TRG in DMEM medium with 5% FBS for 2 h, followed by 675 µM PA treatment for 24 h. The cells were then co-stained with PI and Hoechst 33342 NucBlue Live Cell Stain dye to show the dead and live cells, respectively.

### 2.7. Statistical Analyses

Origin 2019 was used for statistical analysis. The Student’s *t* test or one-way ANOVA was used for two groups or multiple comparisons test. The data was presented as “mean ± s.d.” and *p* < 0.05 was considered as significant.

## 3. Results

**Figure 1:**
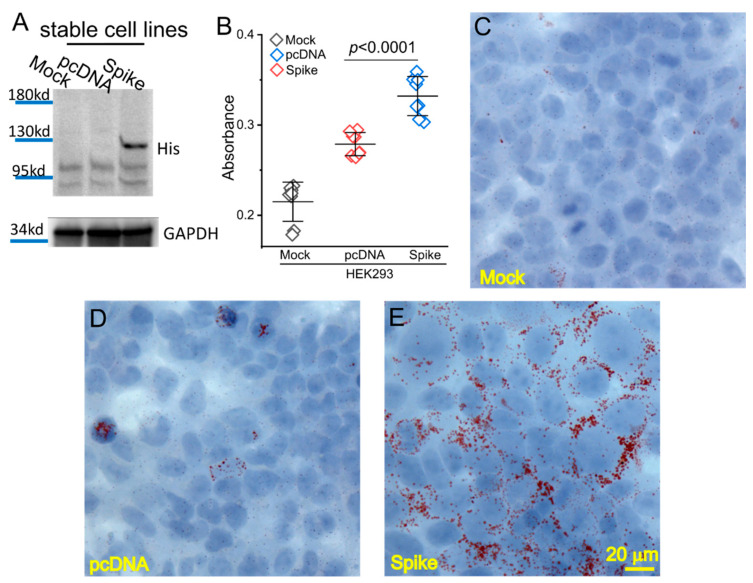
Spike protein caused lipid deposition in host cells. (**A**) Generation of Spike protein stable cell line. The expression of Spike protein was verified using an anti-His tag antibody in the Spike cells. (**B**) The quantification of Oil Red O staining in the mock, pcDNA, and Spike cells with measurement of an absorbance at 492 nm. (**C**–**E**) Histological images showing Oil Red O staining of lipid droplets in the mock (**C**), pcDNA (**D**), and Spike (**E**) cells. Nuclear components were counterstained by hematoxylin.

The stable cell line with expression of the Spike protein was obtained upon neomycin selection after transfection of pcDNA_Spike into HEK293 cells. The control stable cell line with integration of the empty pcDNA was generated simultaneously. The expression of the Spike protein was verified using an anti-His tag antibody (Figure 1A). The same pcDNA_Spike vector was used for production of the Spike protein–pseudotyped (Spp) lentivirus in HEK Phoenix cells in our previous study where the expression of the Spike protein was verified using both anti-Spike S1 and S2 subunit antibodies [24]. We did not observe a significant difference in growth rate between pcDNA and Spike stable cell lines (data not shown). To explore whether the Spike protein had a role in lipid metabolisms in host cells, we performed Oil Red O staining among these cell lines. We found there was a significant accumulation of lipid deposition in the Spike cells compared to pcDNA and mock control cells (Figure 1B). The histological staining showed that the lipid depositions were mainly located on the cell membrane in the Spike cell line (Figure 1C–E), indicating an impairment of lipid metabolism in host cells.

**Figure 2:**
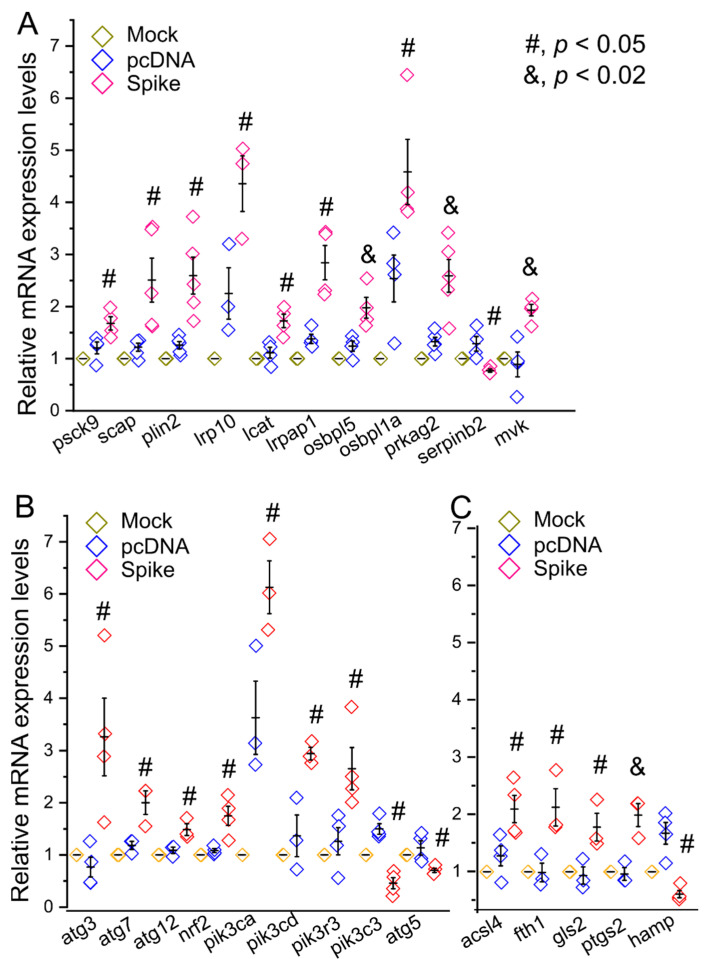
Transcriptional profiles of representative biomarkers for lipid metabolism, autophagy, and ferroptosis. Relative mRNA levels of biomarkers involving lipid metabolism (**A**), autophagy (**B**), and ferroptosis (**C**) in the mock, pcDNA, and Spike cells. The normalization was performed using a converted index of β-actin, nono, and GAPDH as the reference genes.

We next examined transcriptional levels of a panel of 83 genes that are representative markers of lipid metabolism, autophagy, and ferroptosis (Supplementary Table S1). We found the mRNA levels of many lipid metabolic markers were upregulated such as proprotein convertase subtilisin/kexin type 9 (Psck9), SREBF chaperone (Scap), Plin2, low density lipoprotein receptor-related protein 10 (Irp10), lecithin-cholesterol acyltransferase (lcat), low density lipoprotein receptor-related protein associated protein 1 (Irpap1), oxysterol binding protein-like 5 (Osbpl5), oxysterol binding protein-like 1A (Osbpl1a), protein kinase, AMP-activated, gamma 2 non-catalytic subunit (Prkag2), and mevalonate kinase (Mvk); while Serpinb2 was downregulated in the Spike cells (Figure 2A). For the examined autophagic and ferroptotic markers, Atg3, Atg7, Atg12, Nrf2, Phosphatidylinositol-4,5-Bisphosphate 3-Kinase Catalytic Subunit Alpha (Pik3ca), Pik3cd, Phosphoinositide-3-Kinase Regulatory Subunit 3 (Pik3r3), Acsl4, Fth1, glutaminase 2 (Gls2), and Ptgs2 showed an increase in mRNA levels in the Spike cells; while Pik3c3, Atg5, and Hamp showed a decrease in mRNA levels in the Spike cells (Figure 2B,C). These results reveal a negative impact of Spike proteins per se on lipid metabolism, autophagy, and probably ferroptosis in the cell.

**Figure 3:**
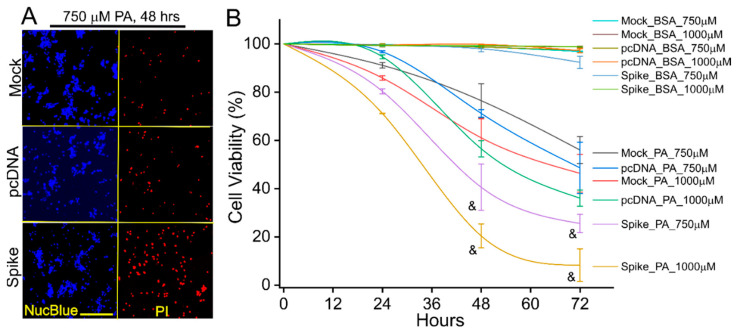
The Spike protein-exaggerated PA-induced lipotoxicity in host cells. (**A**) Fluorescent images showing the cell viability in the mock, pcDNA, and Spike cells treated by 750 µM PA for 48 h in DMEM containing 5% FBS. The cells were co-stained by Propidium Iodide (PI) and Hoechst 33342 NucBlue Live Cell Stain dye to show the dead and live cells, respectively. Scale bar, 25 µm. (**B**) Cell viability curves of mock, pcDNA, and Spike cells in response to PA (750 or 1000 µM) treatments for 24 to 72 h. & indicates *p* < 0.05 in the Spike cells as compared with the mock and pcDNA control cells in response to the same dosages of PA at 48 or 72 h. *n* = 5~6 wells in each dose and time point.

**Figure 4:**
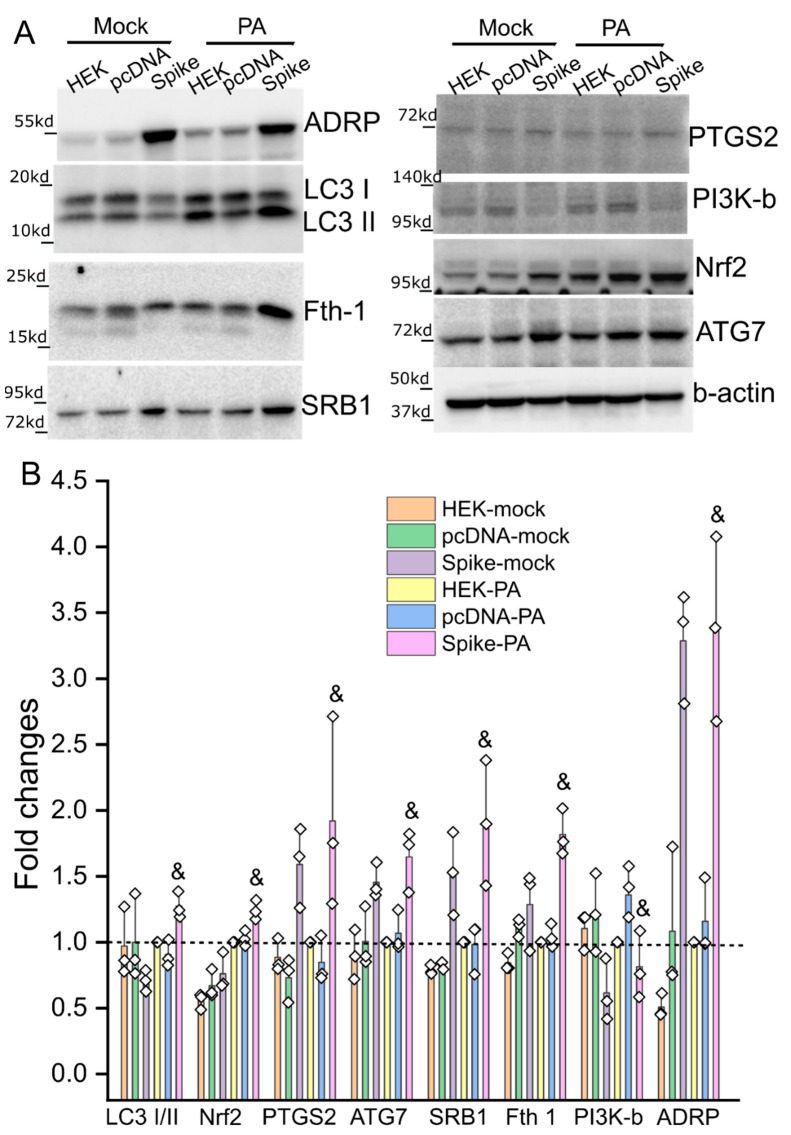
Immunoblot showing the relative changes in levels of some representative biomarkers of impaired lipid metabolism, autophagy, and ferroptosis in response to PA overload. The mock, pcDNA, and Spike cells were treated by 750 µM PA for 24 h in DMEM containing 5% FBS. (**A**) Representative immunoblot showing the protein levels of ADRP, LCI/II, Fth1, Nrf2, PTGS2, PI3K-b, SRB1, and beta-actin in the mock, pcDNA, and Spike cells with or without PA treatment. (**B**) The relative quantification of each biomarker in cells. The protein level for each biomarker was first normalized to the corresponding beta-actin level. The relative ratio of each biomarker within group was then obtained using its’ level in the mock group with PA treatment as 1 (indicated by the dashed line). & indicates *p* < 0.05 in the Spike cells as compared with the pcDNA control cells with the PA treatment. *n* = 3 independent experiments.

We further examined the biological significance of Spike protein expression with a focus on lipotoxicity in vitro. PA overload induced cell death in cultured HNK293 and pcDNA cells (Figure 3 and Figure S1), demonstrating a PA-induced lipotoxicity as described elsewhere [25]. However, the PA-induced cell death was significantly augmented in Spike cells (Figure 3 and Figure S1). The PA-induced cell death also showed a dose- and time- dependent response (Figure 3 and Figure S1). The BSA control did not cause any significant cell death among these cell lines (Supplementary Figure S2). We next examined the expression patterns of some crucial factors in response to PA-induced lipotoxicity. SRB1, ATG7, PTGS2, showed a significantly higher level in the Spike cells than the mock and pcDNA control cells (Figure 4), which were consistent with the changes in mRNA levels (Figure 2). In response to PA treatment, the levels of SRB1, ATG7, PTGS2, LC3 I/II ratio, and Fth1 in Spike cells increased significantly as compared with the pcDNA control cells (Figure 4).

**Figure 5:**
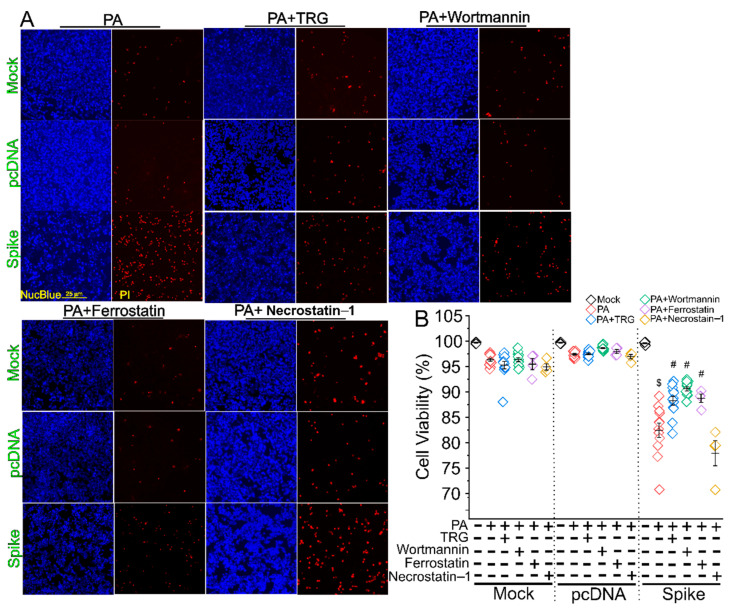
Autophagic/ferroptotic inhibitors attenuated the Spike protein-exaggerated PA-induced necrosis. (**A**) Fluorescent images showing the cell viability in the mock, pcDNA, and Spike cells treated by 750 µM PA treatment for 24 h in DMEM containing 5% FBS with or without a Nrf2 inhibitor TRG, pan-PI3K inhibitor Wortmannin, ferroptosis inhibitor Ferrostain, and apoptotic inhibitor Necrostatin 1. The cells were co-stained by Propidium Iodide (PI) and Hoechst 33342 NucBlue Live Cell Stain dye to show the dead and live cells, respectively. (**B**) Quantitative data showing the cell viability in each treatment group. $ indicates *p* < 0.05 in the Spike cells treated by PA as compared with the Spike cells without PA treatment. # indicates *p* < 0.05 in each group treated with PA and various inhibitors as compared with the Spike cells treated with PA only.

We have previously shown that Nrf2 is the crucial transcriptional factor mediating PA-induced ferroptosis in autophagy-impaired in cardiomyocytes under obese conditions [26]. PI3K has been shown to play a critical role in autophagy [27,28,29]. We then asked whether inhibitors for Nrf2, PI3K, and ferroptosis could attenuate PA-induced lipotoxicity in the Spike cells. Indeed, the Nrf2 inhibitor TRG, PI3K pan inhibitor Wortmannin, and ferroptosis inhibitor ferrostatin, but not a necroptosis inhibitor necrostatin 1, significantly mitigated the PA-induced Spike protein-exaggerated lipotoxicity (Figure 5).

**Figure 6:**
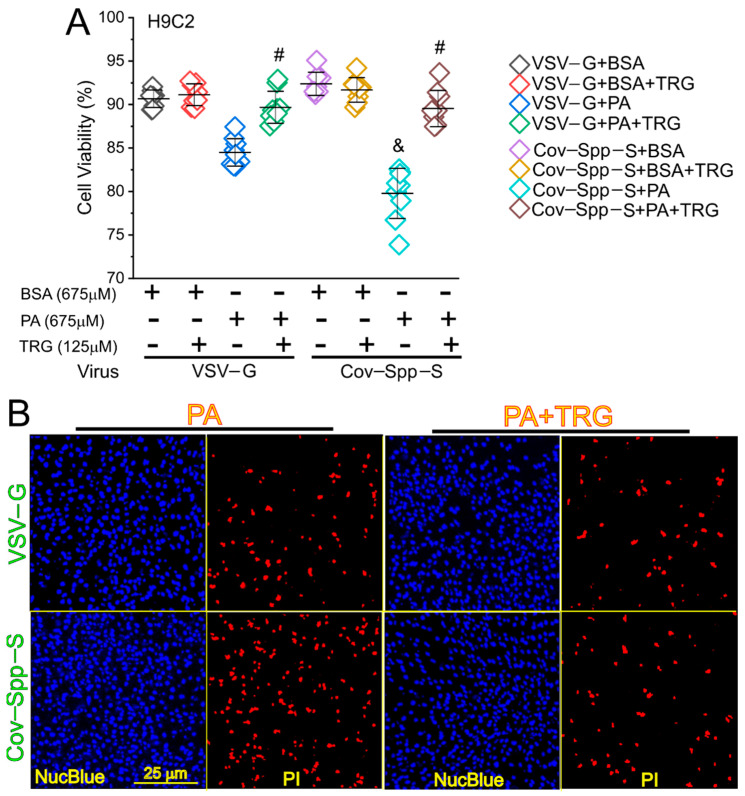
TRG attenuated the Spike protein exaggerated PA-induced necrosis in H9C2 cells. (**A**) Quantitative data showing the cell viability of H9C2 cells after being infected by VSV-G or Cov-Spp-S lentivirus followed by PA treatment with or without TRG. BSA was used as a treatment control. &, indicates *p* < 0.05 in the Cov-Spp-S-infected cells as compared with the VSV-G-infected cells upon PA treatment. #, indicates *p* < 0.05 in the cells treated with PA plus TRG as compared with the cells treated with PA only. (**B**) Fluorescent images showing the cell viability in the VSV-G- or Cov-Spp-S-infected H9C2 cells after PA treatment with or without TRG in DMEM containing 5% FBS. The cells were co-stained by Propidium Iodide (PI) and Hoechst 33342 NucBlue Live Cell Stain dye to show the dead and live cells.

Finally, we attempted to test whether a similar mechanism could be recapitulated in H9C2 cells, a cardiomyocyte-like cell line. We infected the H9C2 cells using CoV-Spp-S lentivirus expressing the Spike protein or VSV-G control virus expressing mCherry. The cells infected by the CoV-Spp-S showed a significantly higher cell death than the cells infected by the VSV-G control virus (Figure 6); the PA-induced Spike protein-exaggerated lipotoxicity could be reversed using the Nrf2 inhibitor TRG (Figure 6).

## 4. Discussion

The data in this study have demonstrated that the Spike protein alone can directly impair lipid metabolic and autophagic pathways in host cells, leading to increased lipotoxicity through ferroptosis. This result has shown a direct and evident role of the Spike protein in exaggeration of pre-existing lipotoxicity, revealing a mechanistic insight into the clinical manifestations of high susceptibility and mortality rate of obese patients with COVID-19. Furthermore, we have shown that the Spike protein-induced necrosis can be suppressed by PI3K pan inhibitor Wortmannin, ferroptosis inhibitor ferrostatin 1, and Nrf2 inhibitor TRG. TRG, an alkaloid enriched in coffee, is among those effective inhibitors, providing a potential and feasible preventive strategy to mitigate COVID-19-associated cardiometabolic pathologies associated with obesity.

Numerous studies have reported lipidomic dysregulation in COVID-19 patients. For example, Shen et al. showed a strong downregulation of over 100 lipids including sphingolipids, glycerophospholipids, fatty acids, and various apolipoproteins in COVID-19 patients [30]. Increased levels of sphingomyelins (SMs), non-esterified fatty acids (NEFAs), and free poly-unsaturated fatty acids (PUFAs) have been shown in COVID-19 patients as well [31,32,33]. Increases in PLA2 activation, which results in long-chain PUFAs, may be associated with the COVID-19 deterioration [34,35]. Our previous studies together with other reports have shown the downregulation of serum LDL-c and HDL-c levels in COVID-19 patients [15,16,17,36]. These lines of accumulated evidence have revealed a central role of lipids and lipid metabolism in the development of COVID-19. In this study, we demonstrate that the Spike protein executes a direct function in altering lipidome via upregulation of a panel of genes involving lipid metabolism and resulting in enhanced lipid deposition on the cell membrane. This data provides direct evidence showing that the Spike protein modulates lipid metabolism in host cells and is an important independent factor contributing to the altered lipidome in COVID-19 patients.

PI3Ks play important roles in autophagy formation during early stages of viral infection for both canonical and non-canonical endocytic pathways [27,28,29]. They are also critical downstream components of growth factor receptor (GFR) signaling cascades, which drive phosphorylation of viral proteins upon SARS-CoV-2 infection [37]. Therefore, this class of enzymes has been proposed as a druggable target for prevention and treatment of SARS-CoV-2 infection. Indeed, inhibition of class I or class III PI3K prevents viral replication [37,38], probably through distinct mechanistic actions on different stages of SARS-CoV-2 viral life cycle. In a distinct mechanism, our data shows that the Spike protein alone can dysregulate expression of various PI3Ks in host cells including upregulation of class I PIK3CA, PIK3CD, and PIK3R3, but downregulation of class 3 PIK3C3, which is required for autophagosome and lysosome fusion [39]. In addition, pan-PI3K inhibitor wortmannin shows a potent inhibition of the Spike-protein exaggerated PA-induced lipotoxicity, suggesting that increasing autophagosome formation while decreasing autophagosome fusion with lysosomes thereby leading to accumulation of autophagosomes may be a cause of Spike protein-exaggerated lipotoxicity. Therefore, targeting of PI3K can be potentially beneficial for those COVID-19 patients with a metabolic precondition of hyperlipidemia.

The transcription factor Nrf2 controls the basal and induced expression of more than 1000 genes in cells that can be clustered into several groups with distinct functions, such as antioxidative defense, detoxification, protein degradation, and iron and lipid metabolism [26]. Thus, the functions of Nrf2 spread rather broadly from antioxidative defense to protein quality control and metabolism regulation. Studies have demonstrated that Nrf2 is required for cardiac adaptation when cardiac autophagy is intact; however, it operates a pathological programme to exacerbate maladaptive cardiac remodeling and dysfunction when myocardial autophagy is inhibited in the settings of sustained pressure overload [40] and chronic type 1 diabetes [21]. Notably, chronic obesity, a pre-type 2 diabetic setting, results in inhibition of myocardial autophagy, thereby leading to cardiac pathological remodeling and dysfunction [41,42]. In this study, the protein level of Nrf2 is upregulated in the Spike cells in response to PA treatment. Furthermore, TRG, a Nrf2 inhibitor, can attenuate Spike-protein-exaggerated PA-induced necrosis in the cell with impaired autophagy. Collectively, it is reasonable to posit a central role of Nrf2 in PA-induced Spike protein-exaggerated lipotoxicity in host cells. However, the detailed pathological mechanism and molecular interactions mediated by impaired Nrf2 pathways need further validation, which will be the goal of our future studies.

There are several limitations for this study, which will be the focus of our future studies. First, the concentrations of PA we used are supraphysiological. The serum average levels of PA range 160 µM in normal subjects and 220 µM in obese, or 160 µM in nondiabetic subjects to 280 µM in diabetic subjects after fast [43,44,45]. We did observe a modest or mild effect at 500 µM and 250 µM after 72 h treatment of PA on the Spike cells (Supplementary Figure S1), indicating that the Spike cells may be sensitive to a physiologically relevant PA concentration after a long-term treatment (>72 h). This possible phenotype shall be investigated in our future studies. Second, the potential effects of other types of fatty acids or lipid species on the Spike cells are not evaluated. In addition, the types of altered lipids or lipid metabolites caused by the Spike protein have not been identified. Third, additional pan-caspase inhibitors are needed to exclude the Caspase-dependent mediated apoptosis in the Spike cells after PA treatment. Fourth, the functional domain(s) of the Spike protein-mediated lipotoxicity and the subcellular locations of expressed Spike are not defined, which can be assessed using a series of constructs to express various truncated Spike mutations. The HEK has very low levels of endogenous ACE2 and TMPRSS2 (www.proteinatlas.org, accessed on 20 May 2020). Therefore, it is unlikely a mechanistic involvement of the signaling pathway associated with a secretion of Spike and binding to the surface ACE2 receptor in our study. Instead, our data demonstrates that the Spike-induced intracellular pathology is largely related to lipotoxicity and impaired autophagy. Fifth, disposition of lipid droplets in the Spike cells shows a predominant cell membrane distribution, indicating that the Spike-mediated lipotoxicity is associated with cell membranes. However, the detailed mechanisms and precise subcellular locations of lipid droplets are yet to be determined and need further investigations. Sixth, this study is the initial step to reveal the pathological role of the Spike-mediated lipotoxicity that is related to autophagy/necroptosis/ferroptosis, indicating a central role of Nrf2. However, the detailed molecular mechanism underlying the Spike protein-induced lipidomic dysregulation is unknown. Our data indicate that Nrf2 may play a central role in the transcriptional level for this pathological process. However, this needs further elucidation and validation using loss-of-function and gain-of-function approaches. Seventh, the current emerging variants, omicron strains, present multiple mutations in the Spike protein; whether these omicron versions of Spike protein variants can enhance or attenuate their functions in lipid metabolic alteration as compared with the alpha version of the Spike protein is unknown. Lastly, the Spike protein-induced impairments in both autophagic and lipid metabolic pathways in host cells are evident. However, whether autophagic impairment is a consequence of or in parallel to lipid metabolic impairments is unknown. Most likely, autophagic impairment is intertangled with lipidomic alterations not only as a result of but also an independent factor for the deterioration in response to lipotoxicity.

In conclusion, this study has demonstrated that the Spike protein can cause lipid deposition and impair lipid metabolic and autophagic pathways in host cells, ultimately leading to increased susceptibility to lipotoxicity via ferroptosis. The Spike protein-enhanced lipotoxicity can be suppressed by the Nrf2 inhibitor TRG, indicating a central role of Nrf2 in COVID-19-associated cardiac complications involving obesity.

## Data Availability

The original data/figures in this study are openly available in https://zenodo.org/ at DOI (10.5281/zenodo.6631465).

## Supplementary Materials

The following supporting information can be downloaded at: https://www.mdpi.com/article/10.3390/cells11121916/s1, Figure S1: PA-induced Spike protein-exaggerated lipotoxicity showed a dose- and time-dependent manner; Figure S2: The BSA with various concentrations doesn’t cause significant cell death among the mock, pcDNA, and Spike cells; Table S1: primer list.
