# Supplementary material for: The Spike Protein of SARS-CoV-2 Impairs Lipid Metabolism and Increases Susceptibility to Lipotoxicity: Implication for a Role of Nrf2
Source: Cells. 2022 Jun 14;11(12):1916. doi: 10.3390/cells11121916 (PMC9221434; doi:10.3390/cells11121916)
Supplement: Supplementary file 1 [file cells-11-01916-s001.zip › cells-1712481-supplementary.pdf]

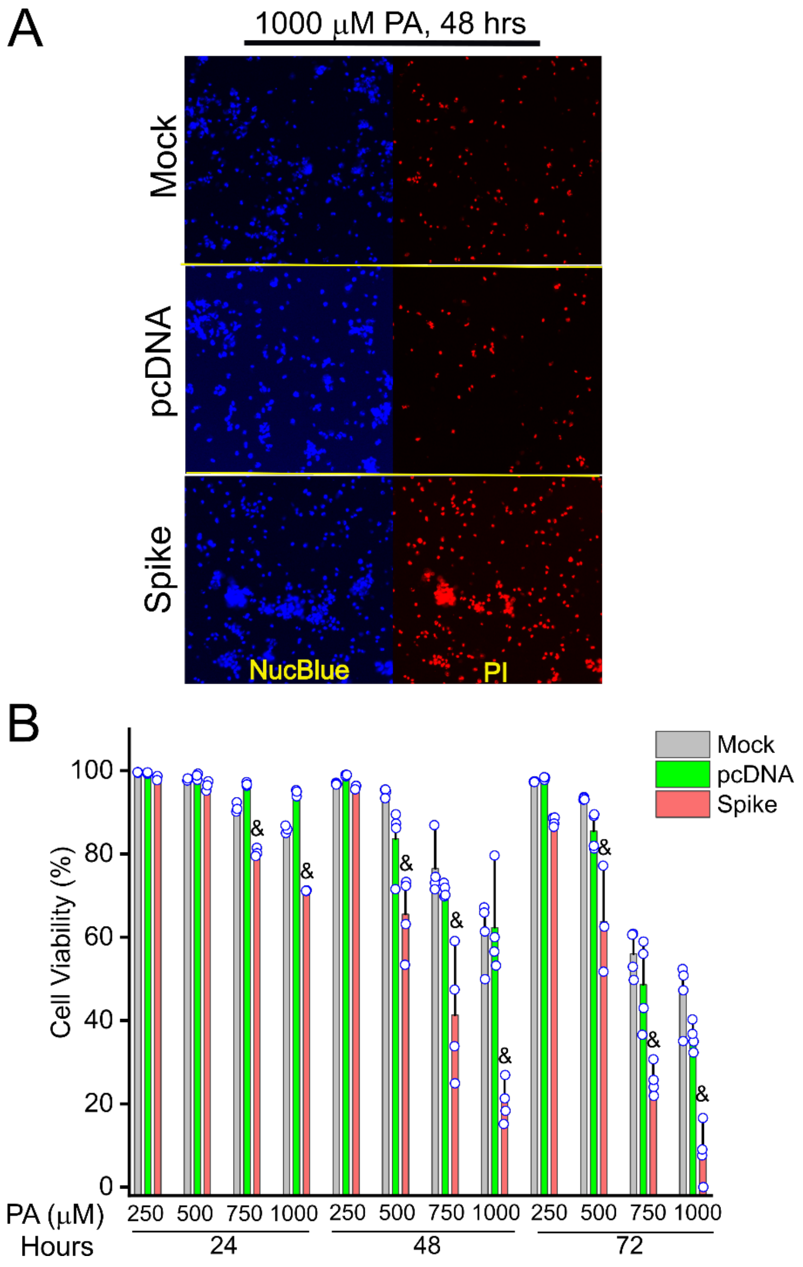

Figure S1. PA-induced Spike protein-exaggerated lipotoxicity showed a dose- and time-dependent manner. &, indicates $p<0.05$ in the Spike cells as compared with the mock and pcDNA control cells for cell viability.

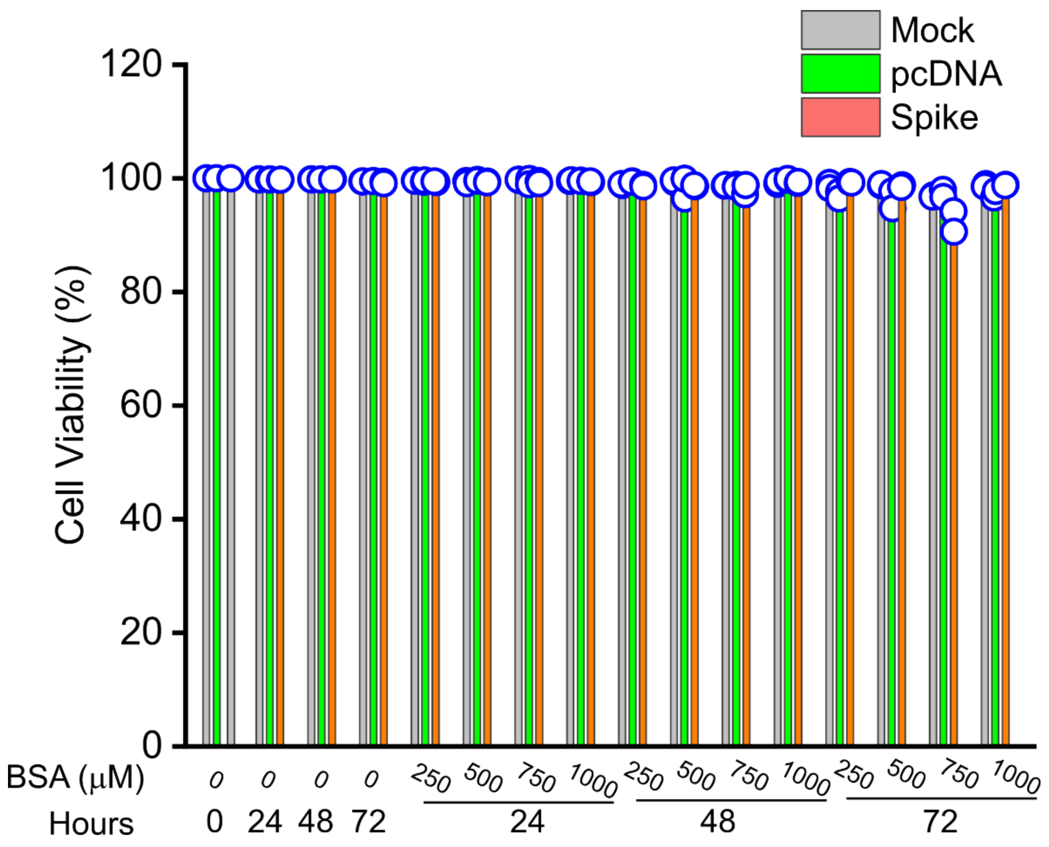

Figure S2. The BSA with various concentrations doesn't cause significant cell death among the mock, pcDNA, and Spike cells.

Table S1. primer list

Gene	Forward	Reverse	Size(bp)
HMGCR	AACAATAAGATCTGTGGTTGGAA	GATGGCTATGCATCGTGTTATTG	102
HMGCS1	AGGAGTAGGACTTGTGCATTC	TCTTGCACCTCACAGAGTATCT	148
HMGCS2	CCAGAATCAGTGGAAGCAAGCT	TGACAGGAAGTCATTGAACATCAG	136
ATF6	CAGGACACATCAGATGGTATTATCC	AAGGAGGTGGAGGAATGTAAAG	121
ATF6B	CCCTGGATGAGGACAACT	CCAATGCCATCCAGAGC	95
ACE	CACAGAGACCAGCAAGATTCT	TTCTGCAACTGGTTCACATCA	105
AGTR1	CGTGACTGTAGAATTGCAGATA	AGAGGATTCAGGCAATTGTT	86
APOB	CCAAAGTACAAAGCCATCACTG	GAAGCATCATCAAGGAAAGTCTG	117
APOE	GACACTGTCTGAGCAGGTGCA	CCTTCAACTCCTTCATGGTCTC	95
AIFM1	CTACAAGCACGCTCTAACATCT	CGTGATCATGGTGCTCTACC	94
APAF1	AAACACCCAAGGTCTCTCTTG	ACTCTTGTCTCTGGTTGTAAGAAG	123
ALOX5	ACTGACGACTACATCTACCTCAG	CACAGTCACGTCGTATGAATCC	67
ATG12	CGAACACGAACCATCCAAGGA	GTTCTGAGGCCACAAGTTTAAG	90
ATG16L1	CACAGAGCAATGTGTAATGAGTG	AGCTCCATCTCTCGAACTATG	93
ATG3	CTCCACGATTATGGTTGTTTGG	CTTCACATGATCCTGACTGATG	113
ATG7	AGAAGAAGCTGAACGAGTATCGG	GCACTGAACTCCAATGTTAAGC	85
ABCA1	TTCCTGATCCTGATCTCTGTTC	CAGAGGGCATGGCTTTATTTG	136
ABCG1	TATGGCTTAGACCGGGAAGA	GTACGATGAAGTCCAGGTACAG	102
BECN1	CTCACAGCTCCATTACTTACCA	CTGGCGAGGAGTTTCAATAAATG	115
CANX	CAAGCATCATGCCATCTCTG	TAGGCACCACCACATTCTATTC	107
CFLAR	GGAGAAGAGTTTCTTGGACCTT	CTGTGGATGTTCTTTAGGCATTT	96
CASP1	CTGCCCACAGACATTCATACA	CACCACTGAAAGAGTGACTTTG	139
CASP2	ATGACGTCCATGTTCTATGTG	GTGCAGGTAACTGTGCAA	92
CASP3	CTCTGGAATATCCCTGGACAAC	ACATCTGTACCAGACCGAGA	88
CASP6	TGGACACCAACATAACTGAGG	CACAGTTTCCCGGTGAGAATA	133
CASP9	CCAGGCAGCTGATCATAGATCT	CCTCTAAGCAGGAGATGAACA	70
CAV1	CAACGATGACGTGGTCAAGA	TCACAGTGAAGGTGGTGAAG	107
CNBP	GGAGAATTCGGACACATTCAAA	ACTTGTCTTGCTGCAGTTGAT	120
CH25H	GTACTTTACACACTGGGACAAA	GCTCAGGTGTTTCTCTTCATTC	93
F3	CATTTCAAAGTGACTAATGCTGATG	TGAAGACCCTGGAGTTCAAA	93
CYP39A1	CTGATATCCACAAGGCCATTA	AGGTCATCCTCAGACACTTTAATC	155
CYP7A1	CAGCGACTTTCTGGAGTTTAT	ACTTCTTCAGTAGCTGCTTTC	62
DFFA	CAGATGGAGGTACAGCTTGGAT	GGCCACATTCTTCCACTTCA	82
ERN1	ACCATCACCATGTACGACAC	GGACACAAAGTGGGACATCT	144
FABP3	TTGTGCGGGAGCTAATTGA	CAGGTCATGCCTCTTTCTCATA	74
FTH1	AAGAGGTGACGGAGGGCTGGCTAT	GTTTCCTGCTTCAACAGTGCT	65
HAMP	CACAACAGACGGGACAACTT	TCCTTCGCCTCTGGAACAT	121
HERPUD1	GTTGGATCACCAATGTCTCA	CAGGCTCCTCTGTGGATTCA	101
IRGM	GGAAGCCATGAATGTTGAGAAA	TCTTCAGAGTCTCCTTGATGTTAG	63
INSIG1	GGAACATAGGACGACAGTTAG	CTCTCAGAATCGGTGGTTT	147

INSIG2	TCATCTTACATGCAGTCTTGGG	AGAGAATGCCATCTCAATACACTC	148
LCAT	AGGTGGATAGCATGGGAGGT	CAGGGCCTCAAGTCAAGGAG	111
LPA	CGTTGCATGTGCCCAGAAAA	TGCTATGAGGGCTCTGTCCT	63
LDLR	ACTGTCTCTGTTGCGGATACC	CCAGTCAGTCCAGTACATGAAG	179
LDLRAP1	AGGATCGTGGCTACAGCTAA	TTGGTGAGGTTGTCTGTCAGGAT	103
LRP10	CTGTGATGGGGTTGATGCCT	CCAAGGTGACATTGCAAGGC	98
LRP12	CAAGAGTGGAAGCAGAATTGT	ACAGAAGCCTGATTAGGTGAAC	126
LRP1B	AACCTGCATTGTGACTGCTGGAAA	CACATTCAACACTTCCATCATCC	132
LRP6	ACAGTCCTTCCACTCATAGGTCA	TAGGAGCATAGTCACTGTCACA	111
LRPAP1	GCCTGATTTAAAGCCAAGAGC	CAGAGGTGAGAGTTGAAAGACG	166
MSR1	CTGATAGCTGCTCCGAATCTGT	CCAATGAGAGGGATGAGAACTG	95
MBTPS1	GCCTAGCCTCAGTCACTCT	GGAGTACCGATGAAGATGGTTTC	74
MBTPS2	CTGGACTGTCGTCTACCTGA	AGATGCTCAGTCCGTTGTTT	144
MVD	TGGCATCGGTGAACAACTT	AGCCACTTCTGAGAGGTCA	133
MVK	GCTCACTCAGCACTTGGTGT	CAGGTACAGATCGGAAGAAAGT	132
NR1H3	AACCCTGGGAGTGAGAGTAT	CATTCATGGCCCTGGAGAA	135
OLR1	TCTGACTCCTGTGAAGCTTCTT	CAAGAGCAAGCAAACCTAACTC	76
OSBPL1A	GCAGTCTCTGGTGAAAGGCT	GCCCAAATGCTCTCCTTCCT	117
OSBPL5	GAGAACCCTGAGGAGTCAGATA	ACATAGGTGGTCCCTCTCC	75
PPIA	TCTTAACCACCAGATCATTCCTTC	CAGCGAGAGCACAAAGATTCTA	96
PLIN2, ADRP	CCTGAAGTCTGTGTGTGAGATG	ACTGCAATTTGCGGCTCTA	119
PPARA	TCATCACGGACACGCTTTC	CATTCGATGTTCAATGCTCCAC	196
PPARD	CACTGAGTTCGCCAAGAG	GCCATACTTGAGAAGGGTAAC	78
PPARG	CAGTGGTTGCAGATTACAAGTATG	GGCTTATTGTAGAGCTGAGTCTT	141
PMVK	TTCAGCGGCAAGAGGAAA	ACAGACATCAGCTCCAAGT	110
PLG	AGAGCCTCTGGATGACTATGT	TCTTCTATACTTCCTGCTCCCA	139
PLAT	GAACAGTCACCGACAACATG	GCCATCGTTCAGACACACCA	121
PLAU	ACCACCAAAATGCTGTGTGC	AGACTCTCGTGTAGACGCCT	82
PCSK9	GGGAAGGACGGCAGATG	TCTTCGGCTGAAACAGATGG	135
PRKAA2	CCAGTGACTGGCAATTACGTG	TCTCTGCTCCACTACTTCATCA	97
PRKAG2	CCTGGATGAGCTTGGAATAGGA	GCAGAGCTGATATTCGTCTTTCC	91
SCARF1	CTGCTCTTCCTGGGCCTTG	CAGACCTGCAGCTTCATCCT	65
SERPINB2	AATGAGGAGGGCACTGAAGC	AGGGTGAGGAAAATCTGCCG	71
SERPINE1	TCAATGACTGGGTGAAGACAC	GCCGTTGAAGTAGAGGGCAT	111
SORL1	ATATCGCCGCAATGTTGTGC	TGGGAGATTGGAGAGGAGGG	119
SCAP	CAGTGCTGTCAAGTGTGTGC	AGCCCATGGTTGTAGAAGGC	145
STARD3	CCACCCCTACTAGCCTGACT	GGCGGAGACAGTCAACTTCA	131
SOAT1	GAGTCTTGTTCTGTCGCCCA	ATCCCAGCACTTTGAGAGGC	120
SREBF1	CTGGTCCGTGCTCAGTACC	TCGCTCTAAGAGATGTTCCCG	82
SREBF2	TTGACTCTGAGCCAGGAAGC	CACAGAAGAATCCGTGAGCG	119
SERP1	TGTGAAGTGACTGACCTTAAGATGT	AGGAAAGTCATTCTGAGGCAGG	145
SYVN1	CAGCTGGTGTTTGGCTTTGA	GTTGTCCCAGGGGTTCTCAC	92

TBP	TCCACAGTGAATCTTGGTTGT	TCCTCATGATTACCGCAGCA	141
THBD	CTAGCTACCTCTGCGTCTGC	GAAACCGTCGTCCAGGATGT	82
TRERF1	GCCAACCAAAACCTGCGAAT	CATGGGCTTCTGAGACAGCA	113
TNFRSF10A	CTGTCCACTTTCGTCTCTGAG	CTGAGACCCTTCAGCTTCTG	145
UBXN4	AGATGGCTGCAAGTTGGGAA	AACTGTAGGCAGGCTTCACT	80
VAMP7	TTTAGGGCAATCGTGTCGCT	ACTACCTCCCTTTGCACAGC	86
VWF	TTGCTGTTATGTAGCCCAGGG	ATTGTGGTGGGAAAGGGAGG	133
ACE2	TGGGACTCTGCCATTTACTTAC	CCCAACTATCTCTCGCTTCATC	105
CYLD	AGCACCACAGCTTCAGATAAA	GGGCAGTTGAGTCTCCAATATAA	93
Nrf2, NFE2L2	TGATTCTGACTCCGGCATTT	GCCAAGTAGTGTGTCTCCATAG	96
NFE2L1	CCTAACTGAGCAGTAGGGATAGA	CTGGCAGGTCACAGAGATAAAT	129
SQLE	GCTTCCTTCCTCCTTCATCAG	CAGTCATTCCTCCACCAGTAAG	72
slc1a5	CCGCTTCTTCAACTCCTTCA	GTAAACCCACATCCTCCATCTC	125
Slc38a1	ACCCTTGTCCCTCTCCTAAA	TTCTGGGTTTCCTTGCTATCC	102
acsl4	GAAGGAGCTGAGGAACCATTAC	CCTCCTGCACAACTGTCAATA	92
GLS	AGGTGGTGATCAAAGGGTAAAG	TCCATGTCCATAGCTGACAAAG	105
GLS2	CTCACCTATGCCATCTCCATAAG	ATTGAGGGAGAGCTTGTTGTAG	102
Got1	GGATGCAGAGAAGAGAGGATTG	GTGGAGGACAACAATGGAGAA	85
Hamp	TCGATCAAAGTGTGGGATGTG	GGGCAGCAGGAATAAATAAGGA	87
TfR	TTTCCACCATCTCGGTCATC	GGGACAGTCTCCTTCCATATTC	102
Ftl	GCTCCCAGATTCGTCAGAATTA	ATAGAAGCCCAGAGAGAGGTAG	107
Fth	GTGCCGTTGTTCAGTTCTAATC	CAAGACAGCCACACCTTAGT	101
Ptgs2	CTGGCGTCCTTTGACTACATT	TCTTGAGTCGCTTGCTGATG	112
ATG5	GATCACAAGCAACTCTGGAT	TTTCTTCTGCAGGATATTCCA	94
LC3	CTTGGGAGTATCACAGGAAA	ACATCTAAACACACAAGGGTA	87
ATG4A	ATACAGTTGCACAGGTGTT	AATGACCACTGTGTTATCC	92
LAMP1	GTGAACTACGACACCAAGAG	TGAGTGTATGTCCTCTTCCAA	148
PIK3C3	AGTGGTGGAACAGATTCACAA	CTTGCTGGTTACTCTGAAGTC	139
ATG4B	ATTGGAGGTGGACACAAAGG	TCTGGTGAATGGAGTAGTAAC	99
PIK3CG	AGTGGTGGAACAGATTCACAA	CTTGCTGGTTACTCTGAAG	128
ATG4C	TTCTCCTGTATTATTGCTTGG	CTGCAATTACGTGATCCTCTA	107
ATG10	TGGAAGAAGATGAGTTCATTG	ACATGTAGCCATCAGAACAGT	129
ATG4D	GGAAGTCTGTGGTCATCCTGGT	GCCAATGAAGTACAGTGAGTG	149
CTSB	ACTTGAAGAGGCTATGTGGT	CTTGCAGGCAGCTTCAGGTC	88
ATG9A	AACCTTGACCTCTTCTTCTCTC	CCGATGAGCATACATGTGAA	71
PIK3C2A	TTAGGCATCTGTGATCGACAC	TCAGAGGTCAGCACAAAAGG	146
PIK3C2B	CCCTGAAGTATGAATGCTACCTG	ATGCTGAACTGAGAGTCCTTG	133
PIK3CA	CCTGCTCATCAACTAGGAAAC	TCAACCACAGTGGCCTTTTTG	76
PIK3CB	CTATCCAGACCAGTACGTTCG	GAGGGCACAATCAAGAAAAGG	130
PIK3CD	AGGAGAATCAGAGCGTTGTG	ACTGAGCATGTGGAAGAGC	146
PIK3R1	CCAACAGGATCAAGTTGTC	AATGCTTCAATAGCTGTCCTT	168
PIK3R2	CGACAAGCGCATGAACAGCCT	CCAGCCACTCGTTGATTTTCT	119
PIK3R3	GAAAGTCGAGATGGAGAGCAG	AGTTCTGTCGAATAGGGCATC	140
PIK3R4	CTTGTATGTGCCAGTTAGGAC	TTGACGAGCTACCACTGTGAT	100
PIK3R5	TGACGCTAAACCTGACAGAAG	CACTTTGATCTGCGATGTGC	89
AKT1	GAGGACGCCAAGGAGATCATG	GCCGTGAACTCCTCATCAAAA	143

AKT2	CCTTGTACCCAATGAAGGAG	TGAATACATCAAGACCTGGAG	71
AKT3	ACAATTCATTCTCTCCTCTTCTT	TCATGTAGATACTCCAGAGG	97
ACTB	GGATCAGCAAGCAGGAGTATG	AGAAAGGGTGTAACGCAACTAA	95
GAPDH	GGTGTGAACCATGAGAAGTATGA	GAGTCCTTCCACGATACCAAAG	122
Nono	AGAGGAGAAGTCGAGGTTAGAG	GCAGAACCCTCTTCAGAGTAATAA	113
